# Spatial representation of serial order in working memory: a cross-cultural comparison between Japanese and Italian adults

**DOI:** 10.1007/s00426-026-02257-x

**Published:** 2026-02-24

**Authors:** Roberta Bettoni, Daichi Yamashiro, Megumi Kobayashi, Masami K. Yamaguchi, Luca Rinaldi, Viola Macchi Cassia

**Affiliations:** 1 Department of Psychology, University of Milano-Bicocca, Piazza dell’Ateneo Nuovo, 1, 20126 Milano, Italy; 2 Department of Brain and Behavioral Sciences, University of Pavia, Pavia, Italy; 3 Research Team for Social Participation and Community Health, Tokyo Metropolitan Institute for Geriatrics and Gerontology, Tokyo, Japan; 4 Department of Psychology, Niigata University, Niigata, Japan; 5 Department of Psychology, Chuo University, Tokyo, Japan

**Keywords:** Serial order, SPoARC effect, Reading/writing habits, Cross-cultural, Horizontal, Vertical

## Abstract

The spontaneous spatialization of serial order in working memory (WM), often reflected by the SPoARC effect, is thought to be influenced by reading/writing habits. However, cross-cultural research comparing the direction of spatial organization in WM remains limited. Here we investigated the spontaneity of this spatialization (its independence from visual-spatial prompts) and its generalizability across different cultural reading systems (Italian vs. Japanese) and spatial axes using a modified SPoARC procedure where visual stimuli are replaced with auditory sequences during encoding, while participants provided manual responses along horizontal (Experiment 1, *N* = 57) and radial (Experiment 2, *N* = 49) axes. We predicted a horizontal left-to-right effect in Italians, a potentially different or absent effect in Japanese due to their mixed reading-writing system, and a top-to-bottom radial SPoARC effect in both groups, considering shared downward information flow experiences. Results confirmed an early-left/late-right horizontal SPoARC effect in Italian readers, which extended, albeit significantly smaller, to Japanese readers. In contrast, no reliable radial spatial bias emerged in either group. These findings are consistent with the view, supported by developmental research, that spatial-order associations emerge from the interaction between early maturational constraints on right hemisphere recruitment for visuo-spatial attention and sensorimotor experience from reading/writing habits.

**Supplementary Information:**

The online version contains supplementary material available at 10.1007/s00426-026-02257-x.

## Introduction

Humans and other animals employ space as an organisational scaffold to retain and manipulate information. The use of a spatially organised mental representation is particularly salient when we deal with numbers, an effect named Spatial-Numerical Association (SNA; Dehaene & Brannon, 2010; Pinel et al., 2004). Beyond introspective reports of mathematicians or ordinary people describing the use of visuo-spatial imagery in basic number representation or complex mathematical reasoning (e.g., Seron et al., 1992), SNAs were first demonstrated in a seminal study reporting several simple reaction time experiments where adults classified visually presented numbers as odd or even using left and right response keys. The main finding was that small numbers, such as 1 or 2, were classified faster with left responses, and larger numbers, such as 8 or 9, were classified faster with right responses (Dehaene et al., 1993). This phenomenon, named as the Spatial Numerical Association of Response Codes (SNARC) after Dehaene et al. (1993), has been consistently replicated in numerous studies across the visual and auditory perceptual domains (see Fischer & Shaki, 2014 for a review).

In the years that followed, research has shown that adults exploit space to represent not only numbers, but also other types of information learnt and stored in long term memory in a conventional fixed order, such as months of the year and days of the week (e.g., Gevers et al., 2004), letters of the alphabet (e.g., Gevers, Reynvoet & Fias, 2003), and temporal events (e.g., Vallesi et al., 2014). Critically, adults would use spatial representations even for newly-learnt information lacking inherent order stored in long-term memory, like lists of abstract figures (e.g., van Opstal et al., 2009) and unrelated words (e.g., Previtali et al., 2010). This has been extended also to information stored in working memory in a seminal study by van Dijck and Fias (2011). The authors instructed participants to memorise arbitrary sequences of visual words indicating fruits and vegetables that were presented in the centre of the screen and to reproduce the sequences in the correct order after a retention interval.

During the retention interval, participants performed a fruit-vegetable classification task by pressing a left- or a right-sided button in response to words that were part of the memorised sequence presented intermixed with new words. Lateralized response times varied systematically as a function of the word’s position in the sequence, with faster left-sided responses for the initial words and faster right-sided responses to the final words, a phenomenon known as Spatial-Positional Association of Response Codes (SPoARC). This evidence, along with related findings (e.g., Gevers et al., 2003; Ginsburg et al., 2017; Previtali et al., 2010) were taken as evidence that spatial-ordinal associations are temporarily constructed during task execution with the serial ordering of information in working memory (WM) when the cognitive system is confronted with a sequence of items processed verbally (Fischer, 2006; Fischer et al., 2010). These associations take the form of an oriented spatial representation which, in people from Western cultures, is organized in a horizontal, left-to-right fashion because of their writing/reading expertise (see Guida & Campitelli, 2019 for a discussion of the expertise account).

While various theoretical accounts have been put forward to explain the SPoARC effect (see review in Hartmann et al., 2021; see also Guida & Campitelli, 2019), the most prominent explanation is that, when confronted with the task of maintaining in memory an arbitrary sequence of items, the brain generates an internal spatial template on which items are translated into directional coordinates, such that spatial attention controls later search and selection (i.e., the mental whiteboard hypothesis; Abrahamse et al., 2014, 2017). In other words, much like when external spatial attention scans the physical environment, the internal equivalent involves shifting the focus of attention across items on the mental whiteboard. Bidirectional interactions between the coding and representation of serial order and spatial attention processes are substantiated by behavioral and electrophysiological evidence. Studies show that retrieving serially ordered verbal items from WM generates electrophysiological responses in the brain that are typically found when shifting visuospatial attention in the external space (Rasoulzadeh et al., 2021) and affects the deployment of both covert (i.e., manual reaction times; e.g., van Dijck et al., 2013) and overt (i.e., oculomotor responses; e.g., De Belder et al., 2015; Rinaldi et al., 2015; Schroth et al., 2025) visuospatial attention resources. The influence of serial position on covert attention is revealed by changes in manual reaction times, as shown by van Dijck et al. (2013). Their work provided evidence that accessing later items in a sequence stored temporarily in WM triggers implicit rightward attention shifts, enhancing dot detection speed on the right side of fixation, when participants are instructed to keep their eyes fixed. Similarly, the link between serial position and overt attention is revealed by changes in explicit oculomotor responses (e.g., De Belder et al., 2015; Rinaldi et al., 2015; Schroth et al., 2025). For example, eye-tracking studies showed that, during serial recall, participants’ eye movements naturally tend to shift to the right as they mentally progress through the sequence (Rinaldi et al., 2015). Moreover, both the direction and size of gaze shifts in physical space are directly associated with the corresponding shifts through serial position in verbal WM (Schroth et al., 2025). Finally, spatial cues targeting overt attention can also affect recall, with leftward cues facilitating initial item retrieval and rightward cues facilitating later item retrieval (De Belder et al., 2015).

The origins of the spatial association of serial positions in WM is still debated (e.g., Guida et al. 2020a, b, c; Nuerk et al. 2015; Patro et al. 2016; van Dijck et al. 2020). However, developmental research suggests that it is influenced by a combination of biological and cultural factors (for reviews, see McCrink & Opfer, 2014; Patro et al., 2016; Rugani & de Hevia, 2017). While to date many of the findings in this area specifically address Spatial-Numerical Associations (SNAs), they are highly relevant because number is a specific case of an ordered sequence, suggesting a shared mechanism for mapping generic ordinal information onto spatial codes. Indeed, the ability to learn sequences and their ordinal structure appears quite early in development (e.g., at birth for magnitudes, Arioli et al., 2025; at 4 months for audiovisual items, Lewkowicz, 2004), and recent evidence suggests that directional spatial cues can enhance this ability (e.g., Bulf et al., 2022; Bulf et al., 2016). For example, studies conducted with 7-month-old infants raised in Western cultures showed that the left-to-right, as opposed to right-to-left, direction of visual item presentation enhances the ability to abstract and generalize increasing (vs. decreasing) numerical order from sequences of non-symbolic numerical displays (Bulf et al., 2022), or repetition-based rule-like structures (e.g., ABA or ABB) from sequences of visual shapes (Bulf et al., 2017).

These findings show that spatial-ordinal mappings are involved during learning of serially ordered visual information from the earliest stages of development, when the ability to retain information in long term memory is still limited and infants lack language and symbolic knowledge. Accordingly, this evidence has been interpreted as suggesting that the early mapping of ordered information into oriented spatial codes reflects a property of serial order WM that is independent of the modality (verbal vs. visual) of the to-be-remembered items, and that language and formal education are not necessary to establishing the directionality of the order-space mapping. Rather, the determinants of its appearance are more likely to be found in the interaction between early maturational constraints and experience with cultural-based directionally-relevant routines through social interactions. Specifically, one prominent hypothesis is that this directionality emerges from the interaction between an early maturational advantage of the right hemisphere in controlling visuo-spatial attention and experience with cultural conventions, such as reading practices (see discussion in Bulf et al., 2016, 2022, and de Hevia et al., 2012). While a direct causal link between early hemispheric asymmetry and spatial biases in human development has yet to be conclusively demonstrated, there is significant evidence supporting its foundational components. Firstly, a maturational advantage of the right hemisphere over the left hemisphere during early development is widely documented (see review in Bisiacchi & Cainelli, 2022). Secondly, this neural asymmetry is paralleled by early behavioral findings. Western 4-month-old infants, just like adults, exhibit a robust leftward bias in the allocation of visuospatial attention (i.e., pseudoneglect) in a paradigm that resembles the line-bisection task - a widely used behavioral measure of relative cerebral asymmetry (Nava et al., 2022). This evidence is particularly relevant as, in adults, pseudoneglect in line-bisection is known to be supported by functional brain lateralization, reflecting the relative primacy of the left visual field and neural activity in the right, contralateral hemisphere. It has been claimed that such early right-hemisphere dominance would determine a leftward asymmetrical exploration of visual space that constrains the structure of infant’s representational space in conjunction with cultural conventions (Bulf et al., 2016; de Hevia et al., 2012). Specifically, passive exposure and active engagement in culturally-driven attentional and behavioral routines during interactions with the caregivers would provide infants with implicit, directionally relevant experience with the dominant direction of their cultural environment (see Göbel et al., 2018 and McCrink et al., 2018 for evidence in toddlers and children).

Although direct published evidence is currently lacking for the role of cultural factors in shaping the direction of early spatial-order association in infancy (but see Macchi Cassia et al., 2020, for preliminary cross-cultural data), research in older children suggests that spatially oriented representations of temporal events (i.e., before-after, Tillman et al., 2022; Tillman et al., 2018) and a SPoARC-like effect for arbitrary sequences start to emerge in some individuals during the last year of kindergarten (i.e. 5 years; van Dijck et al., 2020), and become evident at the group level after children have entered formal literacy education (i.e. 9 years; Guida et al. 2020a, b, c; Moorkens et al. 2025). This supports the notion that the direction of formal writing systems shapes the direction of the mental organization of order in WM. Nonetheless, as in all cases where we question the role of sociocultural experience in shaping cognitive systems, we must turn to cross-cultural research for an answer. Unfortunately, despite evidence of varied space-number associations in numerical processing across cultures with opposing writing directions (e.g., Shaki et al., 2009), studies comparing the direction of serial order spatial organization in WM in individuals from different cultural backgrounds remains limited.

Reading/writing direction has been shown to influence the spatial layouts of time representation across cultures (Fuhrman & Boroditsky, 2010; Vallesi et al., 2014). For instance, English and Hebrew speakers show opposite spatial arrangement of temporal card sequences, and exhibit reversed response-side compatibility effects (the so-called Spatial Temporal Association of Response Codes effect, STEARC; Vallesi et al., 2008) when making rapid temporal order judgments about pairs of pictures, displaying faster ‘earlier’-left and ‘earlier’-right key responses, respectively (Fuhrman & Boroditsky, 2010). Guida et al. (2018), one of the few studies of its kind, reported that similar cross-cultural variations exist in the way space is recruited for the spontaneous internal coding of serial order in WM. Their study examined the existence and the direction of response-side compatibility effects for spatial-positional associations among left-to-right Western readers, right-to-left Arabic readers, and Arabic illiterates. To avoid the triggering of any spatial code, participants memorized random sequences of color patches appearing centrally on the screen, and indicated whether a subsequent probe was part of the memorized sequence using lateralized keys. The results revealed a reversed SPoARC effect in the two literate groups, with right-hand responses faster for earlier items and left-hand responses faster for end items in Arabic readers, and the opposite pattern in Western readers. Illiterates showed no systematic association between the response side and item position.

A recent study by Rasoulzadeh and colleagues (Rasoulzadeh et al., 2024) provided further behavioral and electrophysiological evidence of a right-to-left visuospatial representation of serial information in verbal WM from a population speaking a different right-to-left oriented language, i.e. Iranians speaking Farsi. Despite lacking direct cross-culture comparison, this study replicated Guida et al.’s (2018) results of a reversed SPoARC effect in Arabic readers, and extended behavioral (van Dijck et al., 2013) and electrophysiological (Rasoulzadeh et al., 2021) evidence of spatial attention recruitment during serial order WM retrieval from left-to-right to right-to-left readers. Again, focusing solely on a single culture, Zhou and colleagues (2019) investigated serial order-space interactions in bilingual Chinese participants who, while primarily using left-to-right writing and reading, are also exposed to the traditional top-to-bottom noting system. Notably, the authors investigated the existence and direction of an ordinal position effect for both the horizontal and the vertical spatial dimensions, with the hypothesis that, if the effect is related to the reading/writing direction, it should manifest in this population along both left-to-right and top-to-bottom orientations. Participants were presented with written word sequences appearing centrally on the screen and, after a rehearsal interval, classified memorized words as fruit or vegetable by pressing keys whose orientation (horizontal vs. vertical) was manipulated across experiments. Along with a standard early-left/late-right SPoARC effect in response times, results revealed a bottom-to-top effect, which however was modulated by hand-key associations, as it was only present when the left hand was assigned to the bottom key and the right one to the top key. When they controlled for this left/right hand confound, there was no evidence in favor of a vertical SPoARC effect anymore, suggesting that the finding was driven by an early-left and late-right association of response hands.

Despite the established relevance of the vertical spatial dimension in temporal (e.g., Beracci & Fabbri, 2022, 2024) and numerical order processing (e.g., see review in Winter et al., 2015; Aleotti et al., 2020), where bottom-to-top associations are frequently reported (but see e.g., Dalmaso et al., 2023), the possible vertical orientation of position coding has been significantly overlooked in research focusing on serial order processing. To the best of our knowledge, only a single study has investigated a potential vertical SPoARC effect in Western left-to-right readers. Using the established three-phase (sequence memorization, item classification, sequence recall) procedure established for the SPoARC effect (van Dijck & Fias, 2011), Hartmann and colleagues (2021) measured saccadic eye movements during semantic classification (i.e. consonant vs. vowel) of items from a series of memorized four-letter sequences, with each letter presented centrally on a computer screen. Participants were required to respond by targeting the correct saccade trigger response box by executing leftward and rightward eye movements for half of the trials, and by executing upward and downward eye movements for the other half of the trials. Results revealed that latencies for upward (vs. downward) saccades were relatively shorter when classifying early (vs. late items), suggesting a top-to-bottom orientation of position coding in WM (see also Dutta & Nairne, 1993). The authors interpreted these findings as consistent with the mental whiteboard analogy (Abrahamse et al., 2017), reflecting Westerners’ sensorimotor experience with downward gaze and information flow during reading and writing. Results also suggested a flexible use of spatial frames of references for position coding in right-to-left readers, whereby the spatial format (horizontal or vertical) of the mental representation is determined by specific task demands (i.e., when the task requires horizontal responses, a horizontal association of serial position codes is employed, when the task requires vertical responses, a vertical arrangement is employed).

In conjunction with the results from Zhou and colleagues (2019), these findings point to potential differences in the position coding of serial order information between cultures employing left-to-right reading and those utilizing multiple reading/writing systems with diverse directional orientations. However, along with methodological differences across the studies, the absence of direct cross-cultural comparison leaves these findings inconclusive regarding the influence of reading/writing scanning habits on the observed ordinal position effects in the vertical plane. The current study was designed to address this gap. Specifically, we assessed the existence and direction of the horizontal and vertical SPoARC effects in two groups of participants, left-to-right Western readers, and (bilingual) Japanese readers. The Japanese reading/writing system presents a unique scenario, integrating horizontal and vertical planes, as well as left-to-right and right-to-left directions. While Roman letters and Arabic numerals are conventionally written and read from left to right, top to bottom, traditional Chinese characters (Kanji) and symbols (Hiragana and Katakana) are most often written vertically, with columns running from right to left. Vertical writing is commonly used in street advertising, novels, newspapers and magazines, while horizontal writing is favoured in internet content, textbooks and academic works, and is essential for keyboard use. Consequently, Japanese daily reading/writing practices are characterized by a mix of these directional orientations (Obana, 1997).

We explored spontaneous spatialization of serial order in WM across the two participants’ groups using a modified version of the original procedure established for the SPoARC effect by van Dijck and Fias (2011). Following Guida et al. (2016), to isolate serial order processing from visual spatial attention we replaced written words with auditory stimuli for both the sequence memorization and the item classification phases. Our primary goal in using auditory presentation was to isolate serial order processing from visual-spatial input and any reading-based encoding strategies that might implicitly engender directionality. Guida et al. ([Bibr CR31][Bibr CR33], c; see also Ginsburg et al., 2017 for similar findings) has shown that Western readers flexibly use the external spatial coordinates of items displayed on a screen to build their internal spatial codes for order in WM, as evidenced by reversed SPoARC effects depending on the leftward or rightward direction in which visual letter strings were displayed on the screen during encoding. This suggests that participants used the available spatial information on the screen as positional tags to code order. While many SPoARC studies used visual stimuli presented at the center of the screen (e.g., Ginsburg et al. 2017; Guida et al. 2020a, b, c; van Dijck, 2011), even central visual input risks engaging the visual system in a way that is structurally linked to attention and potentially contaminated by implicit spatial priming related to reading habits, that can be triggered even by single letters or by more complex orthographic characters, as in the case of Japanese. Therefore, to specifically investigate the spatial codes generated spontaneously during retrieval, independent of visual encoding contamination, we opted for auditory stimuli. We did maintain, however, the spatial component of the response selection process from the original procedure, as participants provided manual responses along the horizontal axis in Experiment 1 and the vertical axis in Experiment 2.

The first aim of the study (Experiment 1) was to probe the possible impact of reading habits on the magnitude and/or direction of the horizontal SPoARC effect. While we predicted a left-to-right oriented effect in Western participants, we expected to observe no horizontal spatialization in Japanese participants, due to their mixed reading-writing system. Our second aim (Experiment 2) was to test whether a top-to-bottom oriented SPoARC effect, analogous to that observed for saccadic latencies by Hartmann et al. (2021), would manifest in Western readers using manual responses, and whether this effect would generalize to Japanese participants, considering the shared sensorimotor experience of downward information flow across the two cultures, including both reading and writing and digital media scrolling. Crucially, we note that verticality is often conceptually confounded with radiality or the sagittal dimension in the literature (see Winter et al., 2015). This conceptual overlap is ecologically relevant: when viewing a sheet of paper on a desk, one mentally treats the far (top) edge as higher than the near (bottom) edge, despite both being on the same horizontal plane. This blending of vertical and radial space is typically mirrored in manual response tasks where keys are physically arranged near-to-far on a horizontal surface, specifically to mimic the vertical-sagittal blend that occurs in people’s everyday reading and writing experience. Accordingly, the manual response setup in Experiment 2 physically manipulated the spatial orientation along the front-to-back - i.e. radial - dimension relative to the participant’s body. Therefore, the effect we tested, like that in previous studies using manual responses, should be interpreted as a relationship between serial position and the radial dimension.

## Experiment 1 (Horizontal axis)

### Method

#### Participants

Twenty-eight undergraduate Italian (mean age = 23.82 years, *SD* = 2.88; 19 females; all right-handed) and 29 Japanese (mean age = 20.14 years, *SD* = 1.27; 21 females; all right-handed) students participated in the study. Three additional participants (2 Italian, 1 Japanese) were discarded from the analyses due to chance level performance in the classification task (50%). The Italian participants were recruited from the University of Milano-Bicocca, while Japanese were recruited from Chuo University. None of them reported any visual or linguistic impairment. We conducted an a priori power analysis to determine the required sample size for a repeated-measures ANOVA (group = 2; serial position = 5), assuming a medium effect size (*f* = 0.20), consistent with previous findings (e.g., Zhou et al., 2019) investigating serial order–space interactions in a comparable design (reported effect sizes: 0.017 < *η²ₚ* < 0.022). The analysis indicated that a sample of 48 participants would provide 95% power to detect a significant effect at an alpha level of 0.05 The study protocol was approved by the Ethics Committee of the University of Milano-Bicocca (RM-2020-260), and all participants provided informed consent before their participation.

#### Stimuli

Participants were given 24 WM sequences, each consisting of five unique fruit and vegetable words presented auditorily in their native language (Italian or Japanese) and randomly sampled without replacement from a pool of ten: apple, apricot, carrot, grape, mushroom, orange, pepper, pumpkin, strawberry, and tomato. Auditory stimuli were delivered via professional headphones at a consistent and comfortable volume, with each word and the following interstimulus interval both lasting 1000 ms. Although sequence positions were not perfectly counterbalanced during the memorization phase, random sampling ensured that each word appeared in different positions across participants. In the subsequent classification phase, each word served as a probe 48 times for each participant, and all sequence positions were equally represented across items.

#### Procedure

Participants were tested individually on a computer in the presence of an experimenter in a quiet, dimly lit room. The procedure was modelled after van Dijck and Fias (2011). Participants were given 24 experimental blocks preceded by one practice block, with each block structured into three distinct phases: sequence memorization, item classification, and sequence recall. Each block started with the self-paced successive audio presentation of 5 random words. Participants were instructed to memorize all words in the correct order. Following the final word in the sequence, a rehearsal period of 2500 ms was provided before the classification task started. During the fruit-vegetable classification task, participants underwent 20 trials, each initiated by a 500 ms fixation point, followed by a 1000 ms probe. The central fixation cross served to help participants maintain a stable body/head posture and hand placement relative to the response keys, while also providing a clear visual signal for trial onset without introducing auditory interference. All ten words from the auditory stimuli set were used as probes, each presented twice in a randomized order with the constraint that no word was repeated on consecutive trials. To ensure the memorized sequence was held in working memory during the classification task, participants were instructed to respond solely to the words from the memorized sequence by pressing left (A) or right (L) keyboard keys based on the word’s category. The assignment of response keys to categories was counterbalanced across participants. Responses were required within 2000 ms from probe onset. After this deadline or a response, the screen turned black, followed by a 1000 ms inter-trial interval. Subsequently, a microphone image appeared on the screen, signalling the start of the recall phase. Participants were instructed to verbally recall the entire sequence memorized earlier. The experimenter verified the accuracy of the recalled sequence against the original memorized sequence. Participants received a 30-s break after each recall phase, the experimenter then verbally signalled the beginning of the next block. After completing the 12th block, a long 300-s break was provided. Response speed and accuracy were recorded as dependent variables during fruit-vegetable classification, while accuracy was recorded during the recall phase.

#### Statistical analyses

Data analysis was performed using *R* (R Core Team, 2018) and the *aov* function (Fox & Weisberg, 2019) for Analysis of Variance (ANOVA). Independent sample *t*-tests were conducted to investigate potential group differences in classification and recall accuracy. For each participant, recall accuracy was estimated as the percentage of correct sequences recalled, weighted by the total number of sequences presented (*N* = 24). Classification accuracy and reaction times (RTs) were calculated considering solely go-trials (i.e., those containing a probe that belonged to the memorized sequence) with RTs larger than 100 ms from WM blocks with accurate recall performance.

In line with previous studies investigating the SPoARC effect (e.g., van Dijck and Fias 2011; Guida et al. 2020a, b, c), for each participant we computed RT differences (dRTs) by subtracting the left-hand RTs from the right-hand RTs for each sequence position. Positive dRT indicates faster response with the left hand than the right one, and a negative value indicates the reverse. The Shapiro-Wilk test revealed that dRTs were normally distributed (W = 0.99, *p* = .19). Obtained dRTs were entered as the dependent variable in a 2 × 5 repeated-measures ANOVA with participant group (Italian, Japanese) as between-subject factor and sequence position (1,2,3,4,5) as within-subjects factor. To explore potential further effects within each participant group, we conducted separate repeated-measures ANOVAs, including linear and quadratic components.

Consistent with standard procedure in the SPoARC domain (after Fias et al., 1996), we also computed individual slopes, that is regression coefficients (β values), of the dRT values for each participant following the same rationale as for the RTs. Regression slopes served as indicators of both the strength and direction of the SPoARC effect: a negative slope would reflect a left-to-right bias, where dRTs favor left hand responses at earlier positions, diminishing as the position progresses. Conversely, a positive slope indicates a right-to-left bias. Two-tailed *t*-tests on the regression coefficients were then performed to evaluate whether the obtained regression slopes significantly deviated from zero, and unpaired-sample *t*-tests were used to compare individual regression slopes across the two groups. In addition, to investigate if participants adopted a serial search strategy to retrieve information in WM, we computed mean accurate RTs for each serial position across response sides and entered these values into a 2 × 5 ANOVA with participant group (Italian, Japanese) as the between-subjects factor and serial position (1–5) as the within-subjects factor. We also computed individual regression slopes of mean RTs for each participant, using RTs as the dependent variable and serial position as the predictor, to probe serial scanning of the learned sequences in WM.

Finally, the obtained dRTs slopes of the SPoARC were subsequently correlated with the RTs slopes relating RT to position. By correlating individual RT-by-position slopes (quantifying the degree of serial scanning) with the order-space slopes (quantifying how strongly spatial location was associated with ordinal position), we aimed to determine whether participants showing stronger evidence of sequential scanning in WM also displayed stronger spatialization of serial order (for a similar procedure, see Bottini et al., 2016). A significant correlation would suggest that the spatial effect partly arises from a time-dependent, serial WM retrieval strategy. In contrast, a non-significant correlation would indicate that the observed spatial associations cannot be accounted for by such sequential scanning.

### Results

#### Accuracy

Recall accuracy was similar for Italian (*M* = 77.98, *SD* = 6.60) and Japanese (*M* = 80.52, *SD* = 8.86) participants, *t*(52) = 1.233, *p* = .223, *Cohen’s d* = − 0.325, indicating similar serial memory performance in both groups. Likewise, participants in the two groups performed equally accurate in the fruit-vegetable classification task (Italian: *M* = 81.74, *SD* = 10.1 vs. Japanese: *M* = 82.21, *SD* = 10.3), *t*(55) = 0.177, *p* = .861, *Cohen’s d* = − 0.047, again suggesting similar semantic categorization skills.

#### Reaction times

**Fig. 1:**
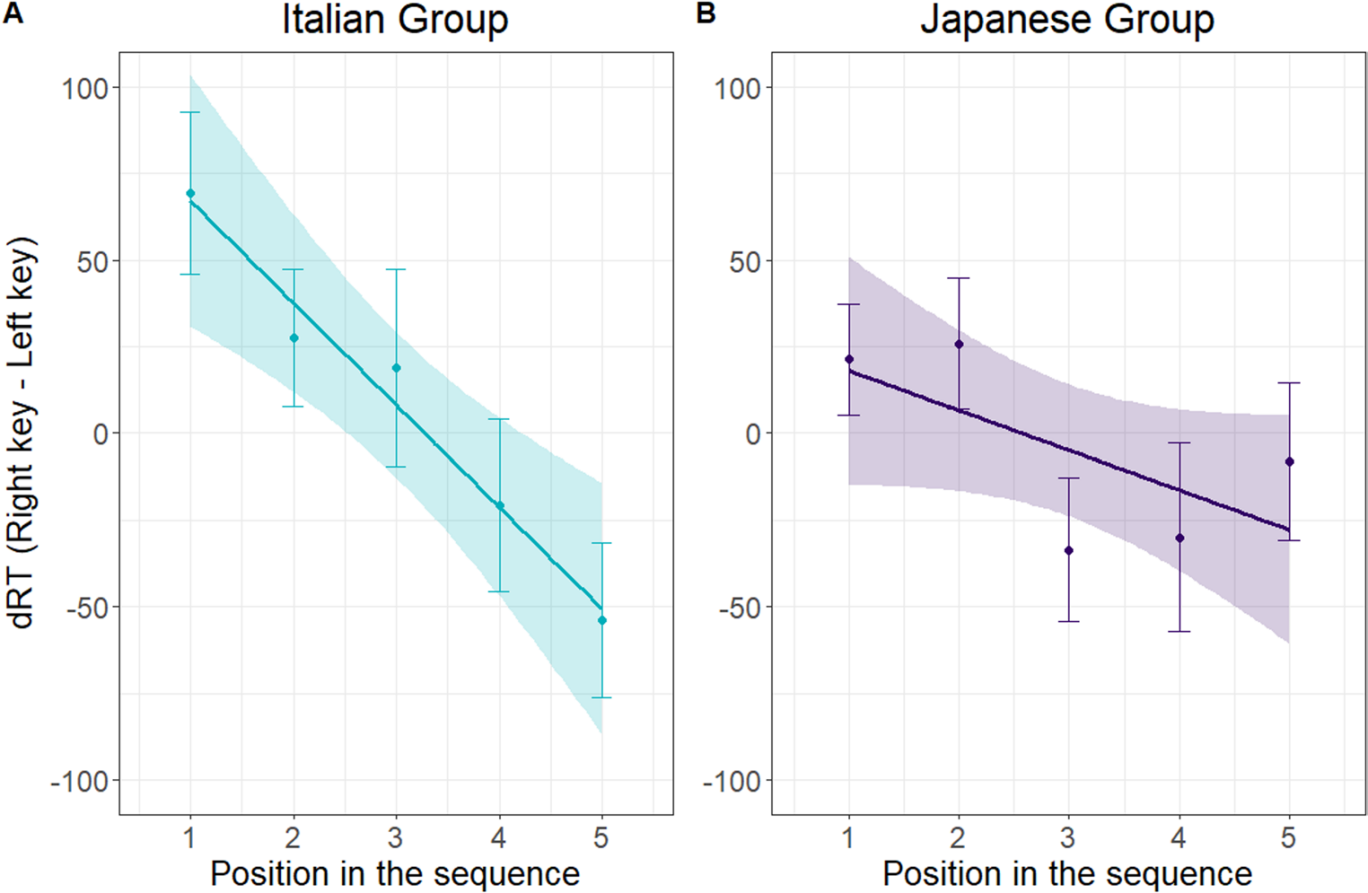
Observed data and regression line representing right-hand reaction times minus left-hand reaction times (dRTs) as a function of the position in the sequence that was probed in the classification trials for Italian and Japanese participants

The ANOVA on dRTs revealed a significant main effect of sequence position, *F*(4,220) = 4.767, *p <* .001, *η*^*2*^_*p*_ = 0.08, indicating faster left sided responses for the initial items and faster right sided responses to the final items. A polynomial contrast confirmed a linear trend *F*(1,220) = 18.039, *p* < .001, suggestive of a left-to-right oriented mapping of serial order into space. The analysis yielded no significant main effect or interaction involving group (*p*s > 0.06). The ANOVA results and polynomial contrasts are reported in the Supplementary Materials (Supplementary Tables S1–S1b), providing full details of main effects, interactions, and estimated marginal means across serial positions. However, as shown in Fig. 1, the patterns of dRTs across item positions in the sequence appeared visibly different for the Italian and Japanese participants. To obtain further insight, we tested the Sequence position main effect for each group with two separate repeated-measures ANOVAs. The main effect proved significant for Italian participants, with polynomial contrast confirming a linear trend, *F*(1,108) = 17.58, *p* < .001, *η*^*2*^_*p*_ = 0.14 and no quadratic trend, *p* = .874. This confirms a significant decrease in the reaction time advantage for the right hand compared to the left as the sequence progresses. The same analysis for Japanese participants showed no significant effect of sequence position, *F*(1,112) = 1.832, *p* = .128.

**Fig. 2:**
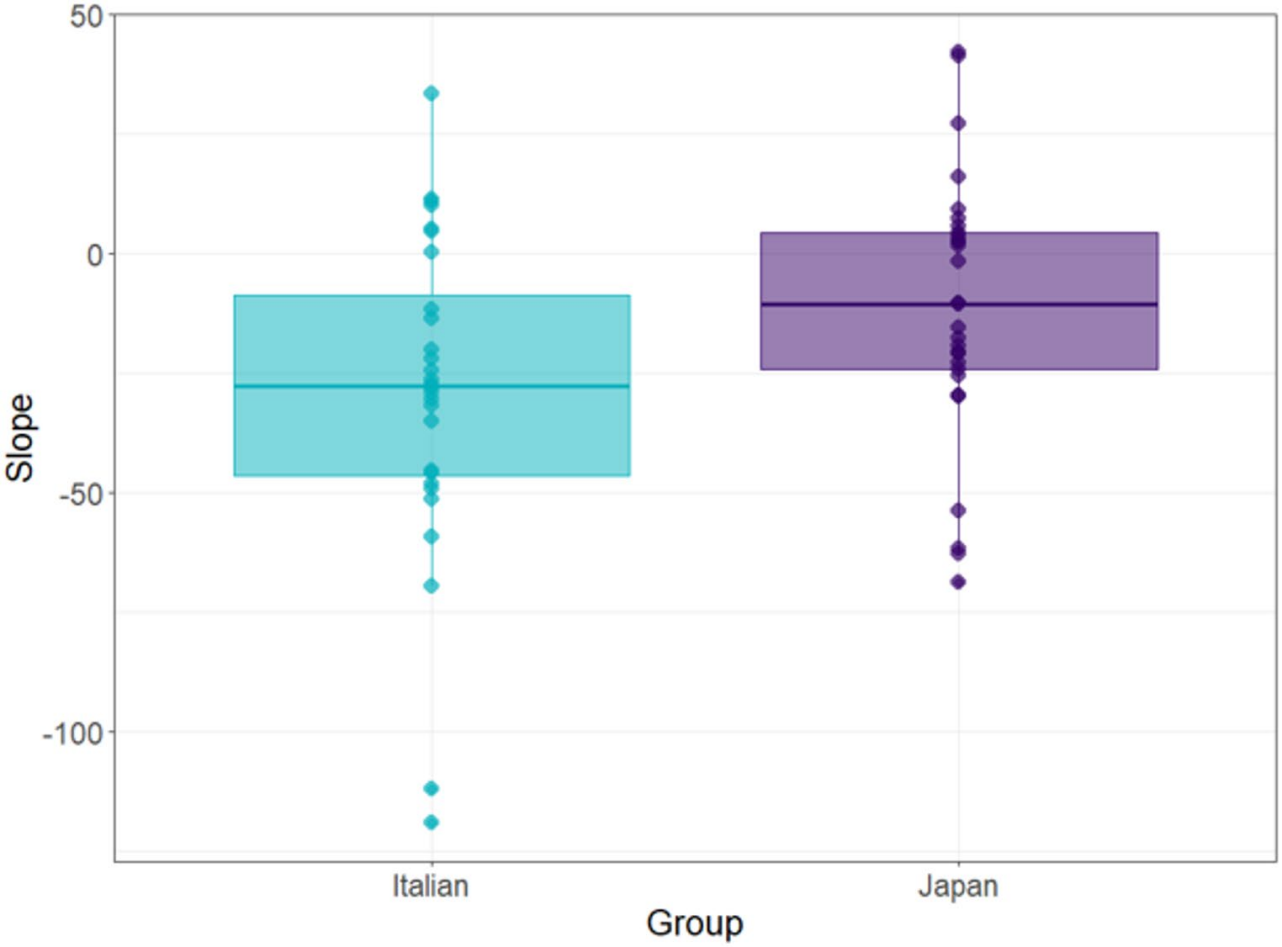
Mean and individual difference reaction time (dRT) slopes for Italian and Japanese participants

Regression analyses on dRTs showed an association of the initial elements with left and the final elements with right in both Italian, *t*(27) = 4.531, *p* <. 001, *Choen’s d* = − 0.856, and Japanese participants, *t*(28) = 2.212, *p* = .035, *Choen’s d* = − 0.412, suggesting a left-to-right SPoARCs effect in both groups. However, the slope of dRTs, reflecting the strength of this effect, differed statistically between the two groups, being significantly steeper in the Italian (*M* = -29.424; *SD* = 34.36) compared to the Japanese group (*M* = -11.453; *SD* = 27.88), *t*(51.98) = 2.164, *p* = .035, *Choen’s d* = − 0.576, indicating a stronger left-to-right spatial-order association in the Italian sample (Fig. 2).

The 2 × 5 repeated-measures ANOVA on mean RTs revealed a significant Serial position main effect, *F*(4,220) = 13.610, *p* < .001, *η*^*2*^_*p*_ = 0.20, indicating that average response latencies increased progressively from the first to the last item position. Polynomial contrasts confirmed a linear trend, *F*(1,220) = 8.014, *p* = .005, *η*^*2*^_*p*_ = 0.20, with no significant quadratic trend, *p* = .375, suggestive of a serial scanning strategy. There were no significant effects involving the Group factor (*p*s > 0.17). The ANOVA results and polynomial contrasts are reported in the Supplementary Materials (Supplementary Tables S2–S2b).

Correlation analyses revealed no significant relationship between the individual slopes of dRTs and the individual slopes relating RT to serial position for either the Italian participants, *r*(26) = 0.16, *p* = .420, or the Japanese participants, *r*(27) = 0.010, *p* = .959. This indicates that the observed association between space and sequence was driven by the position of the items, rather than the elapsed time potentially associated with serial scanning strategies, across all participants.

### Interim discussion

The results of Experiment 1 align with the positional WM account for the SPoARC effect, reinforcing previous evidence that spatial-order associations originate in the positional coding of items in WM. Accordingly, during the item classification task our participants used a serial scanning strategy to access information held in WM, which was organized according to a left-to-right spatial mapping of the items into space.

More crucial to the aim of this study, we obtained no evidence that reading habits influenced the direction of the horizontal SPoARC effect, only its strength. Aggregated analysis revealed similar dRTs (reflecting the left-hand advantage for the initial items and right-hand advantage for the final items) across Italian and Japanese participants. However, the magnitude of the dRT decline across item positions (reflecting the strength of the left-to-right bias) was significantly larger in the Italian than the Japanese group. We propose that this cross-cultural difference in the strength of the horizontal SPoARC effect, but not its direction, might stem from the differing experience with one single mono-oriented reading/writing system in Italians compared to the multiple-oriented systems used by Japanese participants. In Study 2, we investigated whether the SPoARC effect generalizes to the vertical/radial axis in the two participant groups. Based on the common top-down orientation of Western and both traditional and modern Japanese reading/writing systems, we anticipated that, if a SPoARC effect emerged along the radial plane, its strength and direction would be comparable in Italian and Japanese participants. As in Experiment 1, we recorded participants’ lateralized manual responses to auditory sequences, with response keys arranged along the sagittal/radial plane on a horizontal surface. This allowed us to determine whether the vertical/radial SPoARC effect, previously investigated in Western readers with written words (Zhou et al., 2019) or alphabetical letters (Hartmann et al., 2021), would be manifest when serial order stimuli lack external (as with written words) or internal (as with single alphabetical letters) spatial frames, as is the case with auditory sequences.

## Experiment 2 (Radial axis)

### Method

#### Participants

The final sample included 25 Italian students (mean age = 23.16, *SD* = 2.54; 19 females; all right-handed), recruited from the University of Milano Bicocca, and 24 Japanese students (mean age = 20.08, *SD* = 1.35; 13 females; 2 left-handed) recruited from Chuo University. None of them reported any visual or linguistic impairment. Prior to participation, they provided their informed consent to take part in the study, which was approved by the local Ethics Committee.

#### Stimuli and procedure

Experiment 2 employed the same stimuli and procedures as Experiment 1, except the response keyboard in the classification task was rotated counter clockwise by 90°, creating a spatially orthogonal response mapping. Participants again responded only to memorized words by pressing the A or L keys according to the word’s category. On the rotated keyboard, the A and L keys, now positioned close and far from the participant’s body, served as proxies for bottom and top responses, respectively. The mapping of response keys to item categories and left/right hands was counterbalanced across participants.

As in Experiment 1, the experiment consisted of 25 blocks, including one for practice, each comprising a memorization phase, an item classification phase, and a recall phase. A 30-s break followed each block, with the experimenter signalling the next verbally. A longer 300-s break was included after the 12th block. The dependent variables were speed and accuracy in the classification task and accuracy in the recall phase.

#### Statistical analyses

For the radial dimension, we applied the same analysis approach as for the horizontal dimension. To index top-to-bottom and bottom-to-top biases, we calculated the dRT as the difference in mean RTs for responses made with the top versus bottom keys at each serial position in the sequence. A positive dRT value indicates faster response with the bottom key than the top one, and a negative value indicates the reverse. The distribution of dRTs did not significantly deviate from normality (*W* = 0.99, *p* = .26).

Furthermore, given that we counterbalanced across participants the mapping between the hands and the response keys, we conducted a 2 × 2 × 2 × 5 ANOVA on correct mean RTs with serial order position (1,2,3,4,5) and response side (bottom, top) as within-subjects factors, and group (Italian, Japanese) and hand-key mapping (left-top; left-bottom) as between-subjects factors, to assess whether hand position modulated response performance. To further examine the role of hand-key mapping within each group, we additionally conducted separate 2 (response side) x 2 (hand-key mapping) x 5 (serial order position) repeated-measures ANOVAs for the Italian and Japanese samples.

### Results

#### Accuracy

The analysis of recall accuracy, using unpaired *t*-test on the percentage of correctly recalled sequences (out of 24), revealed that Japanese participants (*M* = 80.38, *SD* = 12.10) exhibited superior recall performance compared to Italian participants (*M* = 74, *SD* = 8.61), *t*(47) = 2.123, *p* = .038, *Cohen’s d* = − 0.61. An unpaired t-test on classification accuracy showed no significant difference between Italian (*M* = 82, *SD* = 9.71) and Japanese (*M* = 83.22, *SD* = 11.72) participants, *t*(47) = 0.394, *p* = .696, *Cohen’s d* = − 0.11, indicating similar semantic categorization performance even with a radial response mapping.

#### Reaction times

**Fig. 3:**
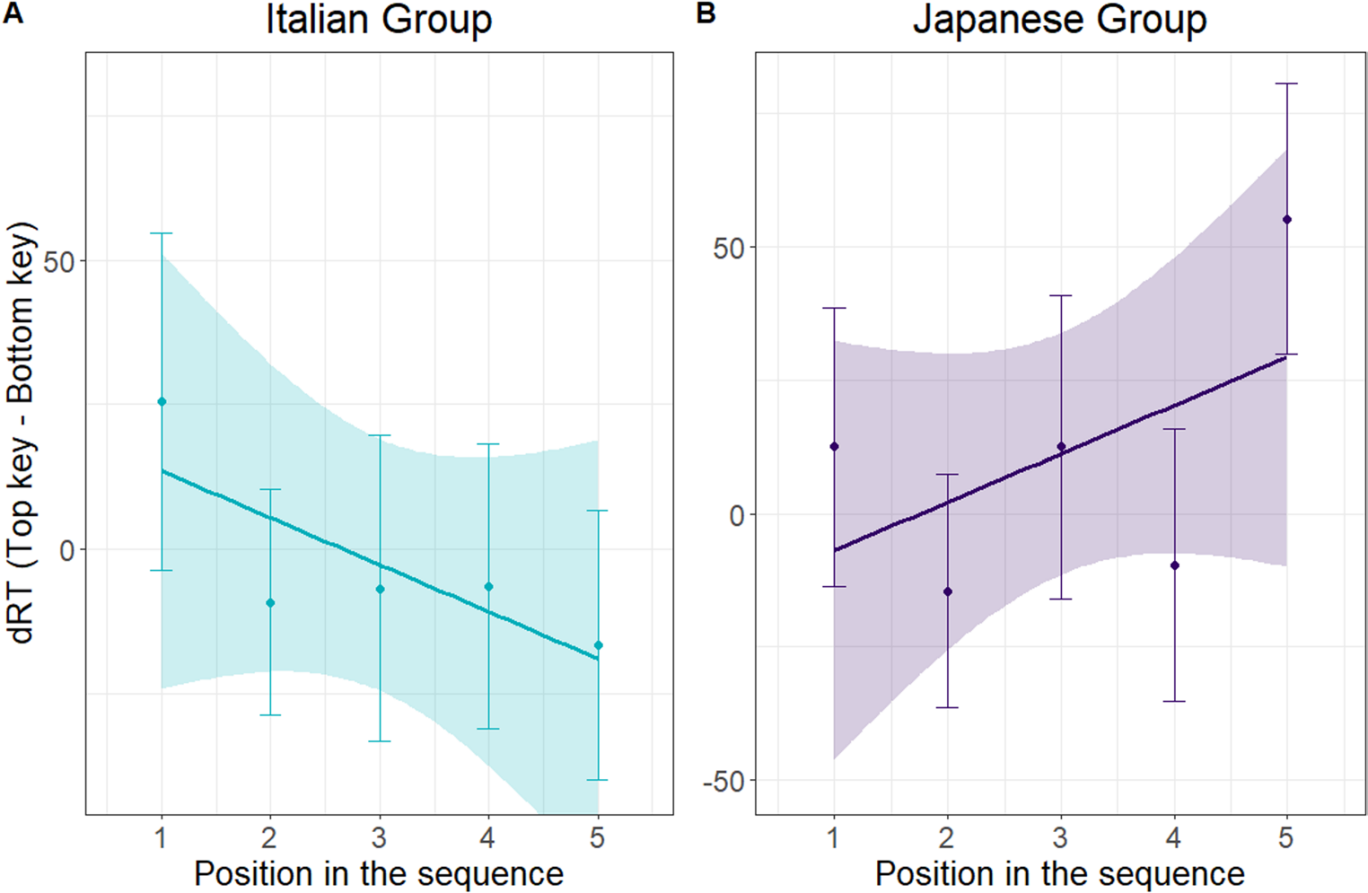
Observed data and regression line representing top-key reaction times minus bottom-key reaction times (dRTs) as a function of the position in the sequence that was probed in the classification trials for Italian and Japanese participants

The 2 × 5 repeated-measures ANOVA on dRTs provided no evidence for a spatial-positional association along the radial plane in either participant group (all *p*s > 0.36; Fig. 3). The ANOVA results are reported in the Supplementary Materials (Supplementary Tables S3–S3b). This lack of association was further confirmed by nonsignificant repeated-measures ANOVAs conducted separately for the Italian, *F*(4,96) = 0.501, *p* = .735, and Japanese, *F*(4,92) = 1.343, *p* = .260, groups.

Results from the linear regression approach also provided no evidence of a radial ordinal position effect. The regression slopes of dRTs did not differ significantly from zero for either the Italian participants (*M* = -8.130; *SD* = 40.76), *t*(24) = 0.997, *p* = .329, Choen’s *d* = − 0.199, and the Japanese participants (*M* = 9.038; *SD* = 39.320), *t*(23) = 1.126, *p* = .272, Choen’s *d* = 0.230. Moreover, the slopes were similar between groups, with no significant difference observed between Italians and Japanese, *t*(47) = 1.501, *p* = .140, Choen’s *d* = − 0.429.

The 2 × 5 ANOVA on mean RTs across response positions revealed a significant effect of serial position, *F*(4,188) = 6.250, *p* < .001, *η*^*2*^_*p*_ = 0.12, indicating that average response latencies increased progressively from the first to the last item position. Despite this overall effect, polynomial contrasts showed no significant linear or quadratic trends, *p*s > 0.12. There were no significant effects involving the factor group (*p*s > 0.21 see Supplementary Materials, Supplementary Tables S4-S4b, for full details on ANOVA results and estimated marginal means). For neither the Italian, *r*(23) = 0.235, *p* = .259, and the Japanese participants, *r*(22) = 0.013, *p* = .953, the slope of dRTs correlated with the slopes of RTs across serial positions, further confirming that neither group spatialized serial order information on a radial/vertical continuum.

The main effect of serial position remained significant in the 2 × 2 × 2 × 5 ANOVA, which included hand-key mapping as an additional between-subjects factor, *F*(4,180) = 6.43, *p* < .001, *η²ₚ* = 0.13. Critically, the three-way interaction between hand-key mapping, sequence position, and response side was not significant, *F*(4,225) = 0.64, *p* = .64, indicating that the SPoARC was not modulated by the hand-key assignment. Moreover, the Group main effect and all interactions involving the Group factor were nonsignificant (all *p*s > 0.21), indicating that the SPoARC effect was consistent across Italian and Japanese participants. Despite the presence of a significant Sequence position x Hand-key mapping interaction, *F*(4,180) = 2.47, *p* = .046, *η²ₚ* = 0.05, post hoc comparisons (Bonferroni-corrected) revealed a consistent pattern across hand-key configurations, with faster responses to the first item compared to the later ones. Specifically, when the left hand was mapped to the top key, RTs were faster at the first position (*M* = 1156.20, *SE* = 33.39) than the third (*M* = 1217.99, *SE* = 33.39), *t*(180) = 3.59, *p* = .004, and fourth (*M* = 1221.37, *SE* = 33.39), *t*(180) = 3.79, *p* = .002, positions. Similarly, when the right hand was mapped to the top, responses were faster at the first position (*M* = 1231.91, *SE* = 33.33) compared to the second (*M* = 1294.31, *SE* = 33.33), *t*(180) = 3.70, *p* = .003, and fifth (*M* = 1288.16, *SE* = 33.33), *t*(180) = 3.33, *p* = .010) positions. These results indicate that there was no significant influence of group or response mapping on the radial/vertical ordinal position effect. Rather, mean RTs reflect a generalized primacy advantage, whereby the first position elicited faster responses than later ones, independently of hand mapping. The ANOVA results including full model statistics and estimated marginal means are reported in the Supplementary Materials (Supplementary Tables S5-S5b). This pattern of results was further confirmed by the 2 × 2 × 5 ANOVAs conducted separately for the Italian and Japanese participants, which confirmed the presence of significant serial order position main effects (*p*s < 0.017) with no significant main effects (Italian: *p* = .152; Japanese: *p* = .422) or interactions (Italian: *p*s > 0.31; Japanese: *p*s > 0.07) involving the hand-key mapping factor. This confirms that performance within both groups was unaffected by the specific hand-key assignment.

## Interim discussion

Both Italian and Japanese participants successfully learned the sequences and accurately categorized the items as vegetables or fruits, with a significant influence of the serial position of the items on their RTs indicating a general slowing as the sequence progressed. Nevertheless, and independent of their reading and writing systems, this influence of serial position on RTs was not modulated by the radial hand-key arrangement along the sagittal/radial plane, as evidenced by the fact that neither the Serial position x Response side nor the Serial Position x Response side x Hand-key mapping interactions reached significance in either groups. This conclusion is further reinforced by the lack of significant effects of serial position on dRTs and by non-significant tests on the dRT slopes, indicating that no radial SPoARC effect emerged.

## General discussion

The present study aimed to investigate whether the reading/writing direction of a culture influences how novel, non-spatial serial order information is spontaneously mentally organized in a spatially defined manner, providing cross-cultural evidence for this relationship. In particular, we chose to investigate this relationship by contrasting the existence and direction of the SPoARC effect along the horizontal and radial axes in two populations that differ in their use of a noting system oriented uniquely along a single axis (left-to-right along the horizontal axis) or that combines different orientations (left-to-right and right-to-left) and different axes (horizontal and radial).

Our results replicated and extended earlier demonstrations of an early-left/late-right horizontal SPoARC effect in Western readers (van Dijck et al., 2013), and showed that the effect generalizes to Japanese readers, albeit with differing strength between the two groups. In contrast, no reliable spatial bias in the organization of serial order along the radial plane was observed in either group.

As our stimuli were auditory, our findings indicate that all participants spontaneously generated horizontally, but not radially, oriented spatial codes without explicit spatial prompts or instructions. This confirms earlier evidence in Western readers that retrieving items at specific positions in auditory sequences presented without visual prompts was accompanied by rightward eye movements along the horizontal axis (Sahan et al., 2022). Our findings extend this evidence to show that this spontaneous horizontal spatialization was more pronounced in participants whose reading/writing habits are consistently and uniquely aligned along the left-to-right direction on the horizontal axis, as seen in our Italian participants showing a magnified left-to-right SPoARC effect.

One possible implication of this finding is that spatialization of serial order in WM arises from the interplay of early maturational constraints (Bisiacchi & Cainelli, 2022) and cultural influences, where culture may intervene in consolidating, weakening or overcoming the initial left-to-right bias observed in preverbal infants (Bulf et al., 2016, 2022; Nava et al., 2022), as seen in, respectively, Western left-to-right readers (van Dijck et al., 2013 and current study), Japanese mixed-readers (current study), and right-to-left Arabic readers (Guida et al., 2018). This could also explain the absence of a vertical/radial bias, even in Japanese participants whose mixed writing system includes top-to-bottom scanning. While Japanese readers likely develop stronger downwards scanning habits than Italians, our results suggest these culture-dependent scanning habits may not be sufficient to counteract the impact of the early leftward visuo-spatial attentional bias resulting from the maturational advantage of the right over the left hemisphere in early ontogeny.

The absence of a vertical/radial bias in our data is consistent with earlier observations by Zhou et al. (2019) in Chinese participants, who presented participants with sequences of written words while using the same manual response settings as in the current study for the critical classification task of items from the memorized sequence. Although Zhou et al. initially reported a bottom-to-top spatial effect (Experiment 2), this pattern was modulated by hand-key associations: when the left hand was assigned to the top key and the right hand to the bottom key (Experiment 3), the vertical SPoARC effect was no longer observed. This suggests that the observed spatial associations did not reflect genuine vertical spatial mappings, but rather arose by response-hand associations. In contrast, our findings are at odds with those obtained by Hartmann and colleagues (2021), who reported a purely vertical and top-to-bottom oriented SPoARC effect in Western readers by recording saccadic eye movements.

As already discussed in Hartmann et al. (2021), this discrepancy can easily be explained by methodological differences, including response settings and stimulus materials, which in the present study were auditory to avoid any spatial prompt. Similar to Zhou et al. (2019), the keyboard was rotated 90° relative to its normal orientation, so that manual responses were mapped to the keys located nearer and farther from the participants’ bodies, which served as proxies for up and down responses, with hand-key mappings counterbalanced between participants. This setup introduced the sagittal (front-back) dimension as the physical spatial manipulation. In fact, while a true vertical axis refers to a top-to-bottom orientation along a vertical plane, where the keys are physically aligned from above to below, our setup along the sagittal axis places the keys on a horizontal plane. Therefore, this configuration may have captured responses along the sagittal axis rather than a true vertical axis, potentially obscuring participants’ spontaneous generation of vertically oriented spatial codes. The existence of multiple spatial mappings for numerical order suggests that a similar complexity might underlie the spatial representation of serial order. Indeed, in the case of numerical order, research has shown that multiple spatial mappings exist, including associations between number magnitude and near/far (sagittal) space (see review in Winter et al., 2015). In some of these studies, Western readers showed distance-based effects by which they associate small numbers with ‘close’ (e.g.,) or ‘back’ (e.g., Hartmann et al., 2012) responses and large numbers with ‘far’ or ‘front’ responses with reference to their body mid-sagittal axis. Other studies using a vertically oriented keyboard on a horizontal plane, similar to our setup and Zhou’s, reported a top-to-bottom ordering of numerical magnitudes in Western and Chinese readers, where the difference between top and bottom RTs decreased as numerical magnitude increased (e.g., Li et al., 2017; Müller & Schwartz, 2007). In addition to showing that vertical SNARC effects can be easily altered by task effector instructions (e.g., Hartmann et al., 2014) and short spatial experience (e.g., Göbel, 2015), this evidence also indicates that the manual response setup used in the current study is indeed sensitive enough to detect biases in spatial-order mappings along the vertical axis. However, our results may indicate that, in the case of serial order, the misalignment between the implicit (i.e. not directly available in the external stimulus) vertical spatial codes and the explicit (i.e., available in the external stimulus) spatial information attached to manual response-keys may have substantially reduced the magnitude of any vertical SPoARC effect.

Another related methodological difference that merits attention as a possible source of inconsistency in the obtained findings relates to the saliency of the vertical spatial dimension for response discrimination in Hartmann et al.’s (2021) study. Participants in that study were asked to classify each centrally-presented letter from the memorized sequence as vowel or consonant by gazing at one of two response boxes displayed at the top and bottom of the screen. Research on the SNARC effect has shown that the impact of the numerical spatial codes on overt behavior varies gradually according to the saliency of the spatial dimensions that is relevant for response discrimination (Gevers et al., 2006). Specifically, in a study by Gevers et al. (2006) subjects had to respond to relatively small and large numbers while the horizontal or vertical response dimension was discriminating whereas the other was not. While a clear SNARC effect was obtained in both dimensions when the response dimension was discriminating, when the dimension was not response discriminating the effect was absent in the vertical dimension and present but smaller in the horizontal dimension. It is therefore possible that, in Hartmann et al.’s (2021) study, the response key’s location on the upper and lower portion of the screen provided salient visual-spatial coordinates that primed participants’ spatial responses according to an implicit vertical positional coding of items in WM.

The experimental setting used in the present study might have also contributed to weakening the strength of the vertical order-space bias, especially in Japanese participants. In contemporary Japanese settings, while street advertising, newspapers, and magazines, as well as traditional formats like manga and novels are read vertically from top to bottom, digital documents and websites favor a horizontal sensorimotor experience, potentially activating left-to-right oriented spatial codes. Because we tested participants while seated in front of a computer screen, a setting more consistent with this dominant horizontal digital reading experience, this context may have suppressed any spontaneous tendency to spatialize along the vertical axis.

Finally, we cannot entirely exclude the possibility that the present study was underpowered to detect subtle vertical spatial biases. While the overall design was adequately powered for the main between-group comparisons testing the impact of directional experience from reading/writing habits, the additional, follow-up analyses targeting specific directional biases within each single group may have been less sensitive. Future research with larger samples could help clarify whether our participants’ failure to show a vertical SPoARC effect reflects a genuine lack of spatial mapping or a power-related limitation.

To conclude, this study further supports the notion that memorizing serial order in working memory involves the use of spatial reference frames. The present findings extend previous research by showing that position coding along the horizontal axis is not exclusive to Western readers but is also present, although significantly weaker, in cultures with a reading/writing system that combines different orientations and dimensions. Our results also suggest that the vertical/radial dimension is less spontaneously activated than the horizontal one in serial order WM tasks, even in cultures with acquired top-to-bottom scanning habits from reading and writing, at least when no direct spatial information is present in the external stimulus or when task demands do not emphasize a purely vertical orientation. Future studies adopting a purely vertical manual response setting - where the response keys are located along a true top-to-bottom axis on a vertical plane - are crucial for a more comprehensive evaluation of how reading and writing habits influence the spontaneous spatialization of serial order in WM along different axes.

## Supplementary Information

Below is the link to the electronic supplementary material.

Supplementary Material 1

## Data Availability

Data that support the findings of this study have been deposited in the Open Science Framework (OSF) and are available upon request: https://osf.io/c56az/?view_only=ce6e151c816e4255ad77a842aafe1275.
