# Supplementary material for: Spatial representation of serial order in working memory: a cross-cultural comparison between Japanese and Italian adults
Source: Psychol Res. 2026 Feb 24;90(2):37. doi: 10.1007/s00426-026-02257-x (PMC12932335; doi:10.1007/s00426-026-02257-x)
Supplement: Supplementary file 1 — Supplementary Material 1 [file 426_2026_2257_MOESM1_ESM.docx]

**Supplementary Materials**

**Spatial representation of serial order in working memory: A cross-cultural comparison between Japanese and Italian adults**

**Short title:** Cross-cultural evidence of spatial-order associations

Roberta Bettoni¹*, Daichi Yamashiro³, Megumi Kobayashi⁴, Masami K. Yamaguchi⁵, Luca Rinaldi²⁺, Viola Macchi Cassia¹⁺

**Affiliations**

¹ Department of Psychology, University of Milano-Bicocca, Milano, Italy
² Department of Brain and Behavioral Sciences, University of Pavia, Pavia, Italy
³ Research Team for Social Participation and Community Health, Tokyo Metropolitan Institute for Geriatrics and Gerontology, Tokyo, Japan
⁴ Department of Psychology, Niigata University, Niigata, Japan
⁵ Department of Psychology, Chuo University, Tokyo, Japan

**Corresponding author:**

Roberta Bettoni
 Department of Psychology, University of Milano-Bicocca
 Milano, Italy
 Email: [inserisci email]

**Contents**

**S1.** Mixed ANOVA - Group × Serial Position on dRTs (Horizontal Axis)

**S2.** Mixed ANOVA - Group × Serial Position on mean RTs (Horizontal Axis)

**S3.** Mixed ANOVA - Group × Serial Position on dRTs (Radial Axis)

**S4.** Mixed ANOVA - Group × Serial Position on mean RTs (Radial Axis)

**S5.** Mixed ANOVA - Group × Hand-Key Mapping × Response Side × Serial Position on mean RTs (Radial Axis)

**S1. Mixed ANOVA: Group x Serial Position on dRTs (Horizontal Axis)**

For the horizontal axis, we conducted a mixed-design ANOVA on dRTs (difference in RTs between right and left-hand responses) with Group (Italian, Japanese) as a between-subjects factor and Serial Position (1,2,3,4,5) as a within-subject factor. Polynomial contrasts (linear and quadratic) were included to describe trends across serial positions. All main effects and interactions are reported in Supplementary Table S1, while estimated marginal means for each serial position are shown in Supplementary Table S1b.

Results showed a significant main effect of Serial Position, indicating that the spatial bias (dRT) varied across positions. No significant main effect of Group or Group × Serial Position interactions were observed.

**Supplementary Table S1 - Repeated-measures ANOVA on dRTs (Total Sample)**

| **Effect** | **Trend** | **Df** | **F** | **p** | **Η²p** |
| --- | --- | --- | --- | --- | --- |
| Group | – | 1, 55 | 0.581 | .449 | 0.01 |
| Serial Position | – | 4, 220 | 4.767 | .001 | 0.08 |
| Serial Position | Linear | 1, 220 | 18.039 | <.001 | – |
| Serial Position | Quadratic | 1, 220 | 0.586 | .445 | – |
| Serial Position × Group | – | 4, 220 | 1.745 | .141 | 0.03 |
| Serial Position × Group | Linear | 1, 220 | 3.540 | .061 | – |
| Serial Position × Group | Quadratic | 1, 220 | 0.964 | .327 | – |

**Supplementary Table S1b - Estimated Marginal Means (EMMs) Across Serial Positions**

| **Serial Position** | **Estimate (dRT)** | **SE** | **95% CI Lower** | **95% CI Upper** |
| --- | --- | --- | --- | --- |
| First | 45.25 | 16 | 13.70 | 76.80 |
| Second | 26.52 | 16 | –5.03 | 58.07 |
| Third | –7.47 | 16 | –39.02 | 24.08 |
| Fourth | –25.36 | 16 | –56.91 | 6.19 |
| Fifth | –31.00 | 16 | –62.55 | 0.55 |

**S2.** **Mixed ANOVA: Group x Serial Position on mean of RTs (Horizontal Axis)**

A similar mixed-design ANOVA was conducted on mean RTs (mRTs) across serial positions (see Supplementary Table S2 for full results, and Supplementary Table S2b for estimated marginal means).

The analysis showed a significant main effect of Serial Position, reflecting systematic changes in overall response speed across positions, but no significant effects of Group or Group × Serial Position interactions were observed.

**Supplementary Table S2 - Repeated-measures ANOVA on mean of RTs (Total Sample)**

| **Effect** | **Trend** | **Df** | **F** | **p** | **η²p** |
| --- | --- | --- | --- | --- | --- |
| Group | – | 1, 55 | 0.125 | .725 | .002 |
| Serial Position | – | 4, 220 | 13.610 | <.001 | .20 |
| Serial Position | Linear | 1, 220 | 8.014 | .005 |  |
| Serial Position | Quadratic | 1,220 | 0.789 | .375 |  |
| Serial Position × Group | – | 4, 220 | 1.628 | .168 | .03 |
| Serial Position × Group | Linear | 1, 220 | 0.084 | .772 |  |
| Serial Position × Group | Quadratic | 1, 220 | 1.683 | .202 |  |

**Supplementary Table S2b - Estimated Marginal Means (EMMs) Across Serial Positions**

| **Serial Position** | **Estimate (mRT)** | **SE** | **95% CI Lower** | **95% CI Upper** |
| --- | --- | --- | --- | --- |
| First | 1186 | 22.4 | 1141 | 1230 |
| Second | 1253 | 22.4 | 1209 | 1298 |
| Third | 1235 | 22.4 | 1191 | 1280 |
| Fourth | 1248 | 22.4 | 1203 | 1292 |
| Fifth | 1249 | 22.4 | 1204 | 1293 |

**S3.** **Mixed ANOVA: Group x Serial Position on dRTs (Radial Axis)**

For the radial axis, we conducted a 2 x 5 repeated-measures ANOVA on dRTs, with Group as between-subjects factor and Serial Position (1,2,3,4,5) as within-subject factor (see Supplementary Table S3 for full results, and Supplementary Table S3b for estimated marginal means).

No significant main effects or interactions were observed, so polynomial contrasts were not computed.

**Supplementary Table S3 - Repeated-measures ANOVA on dRTs (Total Sample)**

| **Effect** | **Df** | **F** | **P** | **η²p** |
| --- | --- | --- | --- | --- |
| Group | 1, 45 | 0.491 | .487 | .01 |
| Serial Position | 4, 180 | 0.766 | .549 | .02 |
| Serial Position × Group | 4, 180 | 1.091 | .362 | .02 |

**Supplementary Table S3b — Estimated Marginal Means (EMMs) Across Serial Positions**

| **Position** | **Estimate (dRT)** | **SE** | **95% CI Lower** | **95% CI Upper** |
| --- | --- | --- | --- | --- |
| First | 18.82 | 17.8 | -16.3 | 53.9 |
| Second | -12.01 | 17.8 | -47.1 | 23.1 |
| Third | 2.66 | 17.8 | -32.5 | 37.8 |
| Fourth | -8.20 | 17.8 | -43.3 | 26.9 |
| Fifth | 19.18 | 17.8 | -15.9 | 54.3 |

**S4.** **Mixed ANOVA: Group x Serial Position on mean of RTs (Radial Axis)**

A 2 x 5 mixed-design ANOVA was conducted on mean RTs (mRTs) along the radial axis, with Group as a between-subjects factor and Serial Position as a within-subject factor. Polynomial contrasts (linear and quadratic) were included (see Supplementary Table S4 for full results, and Supplementary Table S4b for estimated marginal means).

The analysis revealed a significant main effect of Serial Position, indicating changes in response speed across positions, but no significant main effects or interactions involving Group were found.

**Supplementary Table S4 - Repeated-measures ANOVA on mean of RTs (Total Sample)**

| **Effect** | **Trend** | **Df** | **F** | **p** | **η²p** |
| --- | --- | --- | --- | --- | --- |
| Group | – | 1, 47 | 1.60 | .213 | .03 |
| Serial Position | – | 4, 188 | 6.25 | .001 | .12 |
| Serial Position | Linear | 1, 188 | 2.42 | .122 | – |
| Serial Position | Quadratic | 1, 188 | 1.49 | .224 | – |
| Serial Position × Group | – | 4, 188 | 1.12 | .350 | .02 |
| Serial Position × Group | Linear | 1, 188 | 2.46 | .119 | – |
| Serial Position × Group | Quadratic | 1, 188 | 0.43 | .515 | – |

**Supplementary Table S4b — Estimated Marginal Means (EMMs) Across Serial Positions and Groups**

| **Position** | **Estimate (mRT)** | **SE** | **95% CI Lower** | **95% CI Upper** |
| --- | --- | --- | --- | --- |
| First | 1194.18 | 24.58 | 1144.76 | 1243.60 |
| Second | 1245.45 | 23.45 | 1198.31 | 1292.59 |
| Third | 1238.50 | 23.67 | 1190.91 | 1286.08 |
| Fourth | 1241.82 | 22.90 | 1195.77 | 1287.87 |
| Fifth | 1242.88 | 24.67 | 1193.28 | 1292.49 |
|  |  |  |  |  |

**S5. Mixed ANOVA: Group × Hand–Key Mapping × Response Side × Serial Position on mean of RTs (Radial Axis)**

To evaluate whether hand–key mapping influenced performance, a 2 x 2 x 2 x 5 repeated-measures ANOVA was conducted on mean RTs (mRTs) with Serial Position (1,2,3,4,5) and Response Side (bottom, top) as within-subjects factors, and Group (Italian, Japanese) and Hand–Key Mapping (left-top, left-bottom) as between-subjects factors. All main effects and interactions are reported in Supplementary Table S5, and estimated marginal means for each condition are shown in Supplementary Table S5b.
The analyses revealed no significant main effect of Hand–Key Mapping and no significant interactions involving this factor. Follow-up analyses conducted separately for each group confirmed that Hand–Key Mapping did not significantly affect performance.

**Supplementary Table S5 - Repeated-measures ANOVA on m RTs (Total Sample)**

| **Effect** | **Df** | **F** | ***p*** | ***η²p*** |
| --- | --- | --- | --- | --- |
| Group | 1, 45 | 1.61 | .211 | .03 |
| Hand-Key Mapping | 1, 45 | 2.44 | .126 | .05 |
| Group × Hand-Key Mapping | 1, 45 | 0.06 | .807 | .001 |
| Serial Position | 4, 180 | 6.43 | < .001 | .13 |
| Serial Position × Group | 4, 180 | 1.15 | .335 | .02 |
| Serial Position × Hand-Key Mapping | 4, 180 | 2.47 | .046 | .05 |
| Serial Position × Group × Hand-Key Mapping | 4, 180 | 0.91 | .462 | .02 |
| Response Side | 1, 225 | 0.26 | .610 | .001 |
| Serial Position × Response Side | 4, 225 | 0.65 | .625 | .01 |
| Group × Response Side | 1, 225 | 0.76 | .386 | .003 |
| Hand-Key Mapping × Response Side | 1, 225 | 0.66 | .418 | .003 |
| Serial Position × Group × Response Side | 4, 225 | 0.93 | .447 | .02 |
| Serial Position × Hand-Key Mapping × Response Side | 4, 225 | 0.64 | .635 | .01 |
| Group × Hand-Key Mapping × Response Side | 1, 225 | 2.89 | .090 | .01 |
| Serial Position × Group × Hand-Key Mapping × Response Side | 4, 225 | 0.39 | .814 | .007 |

**Supplementary Table S5b — Estimated Marginal Means (EMMs)**

| **Hand-Key Mapping** | **Response Side** | **Serial Position** | **Estimate (mRT)** | **SE** | **95% CI Lower** | **95% CI Upper** |
| --- | --- | --- | --- | --- | --- | --- |
| left-top | bottom | 1 | 1151.96 | 35.76 | 1080.69 | 1223.22 |
| left-top | bottom | 2 | 1214.14 | 35.76 | 1142.88 | 1285.41 |
| left-top | bottom | 3 | 1224.36 | 35.76 | 1153.09 | 1295.62 |
| left-top | bottom | 4 | 1219.30 | 35.76 | 1148.04 | 1290.57 |
| left-top | bottom | 5 | 1185.38 | 35.76 | 1114.12 | 1256.65 |
| left-top | top | 1 | 1160.45 | 35.76 | 1089.19 | 1231.72 |
| left-top | top | 2 | 1178.70 | 35.76 | 1107.44 | 1249.96 |
| left-top | top | 3 | 1211.62 | 35.76 | 1140.36 | 1282.88 |
| left-top | top | 4 | 1223.43 | 35.76 | 1152.17 | 1294.70 |
| left-top | top | 5 | 1208.93 | 35.76 | 1137.67 | 1280.20 |
| left-bottom | bottom | 1 | 1217.33 | 35.61 | 1146.35 | 1288.31 |
| left-bottom | bottom | 2 | 1288.79 | 35.61 | 1217.81 | 1359.77 |
| left-bottom | bottom | 3 | 1249.49 | 35.61 | 1178.51 | 1320.47 |
| left-bottom | bottom | 4 | 1272.97 | 35.61 | 1201.99 | 1343.95 |
| left-bottom | bottom | 5 | 1280.10 | 35.61 | 1209.12 | 1351.09 |
| left-bottom | top | 1 | 1246.50 | 35.61 | 1175.52 | 1317.48 |
| left-bottom | top | 2 | 1299.83 | 35.61 | 1228.85 | 1370.81 |
| left-bottom | top | 3 | 1268.38 | 35.61 | 1197.40 | 1339.36 |
| left-bottom | top | 4 | 1253.45 | 35.61 | 1182.47 | 1324.43 |
| left-bottom | top | 5 | 1296.21 | 35.61 | 1225.23 | 1367.19 |
